# Long COVID y salud oral: ¿estamos listos?

**DOI:** 10.21142/2523-2754-1003-2022-114

**Published:** 2022-09-28

**Authors:** Yalil Augusto Rodríguez Cárdenas

**Affiliations:** 1 División de Radiología Oral y Maxilofacial, Universidad Nacional de Colombia. Bogotá, Colombia. yalilrodriguez@gmail.com Universidad Nacional de Colombia División de Radiología Oral y Maxilofacial Universidad Nacional de Colombia Bogotá Colombia yalilrodriguez@gmail.com

Según recientes reportes epidemiológicos, el síndrome respiratorio agudo severo por coronavirus 2 (SARS-CoV-2) pandémico está próximo a desaparecer o, por lo menos, a que tengamos que convivir con él en forma de endemia, debido, en parte, a la inmunidad generada por las vacunas y, por otro lado, a la inmunidad de rebaño. Mientras esto sucede, el mundo espera nervioso la aparición de nuevas variantes o enfermedades zoonóticas, como la reciente viruela símica.

A todo este cuadro se suma la aparición de la llamada Enfermedad Prolongada del COVID, o *Long COVID*, cuyos numerosos síntomas están comenzando a estudiarse en todo el mundo. Colectivamente, los investigadores ya han avanzado en la identificación de los mecanismos que subyacen a esta afección, conocida formalmente como secuelas de la COVID aguda (PASC). Algunos estudios han identificado hasta 200 manifestaciones relacionadas con ella, y las más comunes son la fatiga, la dificultad para respirar y el sentido del olfato disfuncional ^1^. Sin embargo, identificar rápidamente esta condición muchas veces es una tarea compleja. Algunos estudios estiman que del 30% al 70% de los pacientes reportarán síntomas de 12 a 24 semanas después de la infección ^2^^,^^3^. Esto hace difícil su análisis y estudio.

En lo que tiene que ver con la odontología, muchos pacientes han volcado sus preguntas hacia nuestra profesión, en busca de posibles respuestas y relaciones con estos síntomas y manifestaciones. Sobre las relaciones entre *Long COVID* y salud oral, la literatura es aún escasa, y es nuestro deber estar atentos a las últimas evidencias que la ciencia nos proporciona. Un grupo de investigación evaluó a una población sudafricana y reportó a la gingivitis y la periodontitis como comorbilidades asociadas al PASC ^4^. Una serie de casos reportan manifestaciones orales especiales, como exceso de saliva ^5^. Además, en un reporte de caso de un individuo de 77 años, los autores reportan lesiones herpéticas orales y fúngicas recurrentes ^6^. Sin embargo, aparentemente, las respuestas y manifestaciones orales podrían ser mucho más amplias y numerosas de las que la literatura científica reciente ha reportado. 

En una breve encuesta telefónica realizada entre colegas, los cuadros clínicos que con frecuencia han encontrado en pacientes diagnosticados con PACS han sido la presencia de aftas y estomatitis herpética de resolución lenta, así como inflamación de las glándulas salivares y de la lengua en la mayoría de ellos. Estas dos últimas manifestaciones han sido incluidas en una interesante revisión, en la cual dividen en 4 grupos las manifestaciones de PACS según su impacto. Cabe señalar que en nuestro análisis no se ha incluido a las disfunciones de la ATM, mencionadas por casi todos los colegas, principalmente por ser un cuadro con etiología multifactorial y, lo que es más importante, ante estos cuadros clínicos necesariamente deben surgir numerosas preguntas de investigación con sus correspondientes hipótesis. Todo un banquete para los investigadores.

Esta es solo una pequeña parte de lo que enfrentaremos en un futuro, pero que está presente. Las preguntas que surgen entonces son las siguientes: ¿estamos preparados para enfrentarlo? ¿Somos capaces de despejar estas dudas a un paciente con *Long COVID*? Para la odontología, supone un reto mantener la salud oral en este tipo de pacientes, pues el número de covariables presentes es amplio y aún desconocido, pero plantea un interesante reto para nuestra profesión, nos provee un nuevo campo de investigación y, consecuentemente, nos lleva a una mayor discusión.
